# Sorption Capacity of AlOOH/FeAl_2_ Composites towards As(V)

**DOI:** 10.3390/ma16176057

**Published:** 2023-09-04

**Authors:** Sergey O. Kazantsev, Konstantin V. Suliz, Nikolay G. Rodkevich, Aleksandr S. Lozhkomoev

**Affiliations:** Institute of Strength Physics and Materials Science of the Siberian Branch of the Russian Academy of Sciences, pr. Akademicheskii 2/4, 634055 Tomsk, Russia; kzso@ispms.ru (S.O.K.); konstantin.suliz@gmail.com (K.V.S.); ngradk@ispms.ru (N.G.R.)

**Keywords:** bimetallic nanoparticle, iron, arsenic adsorption, boehmite, nanostructures, oxidation, composites

## Abstract

The treatment of wastewater from arsenic compounds is an important and urgent problem. Composite nanostructures consisting of boehmite and iron compounds have a high adsorption capacity towards As(V) specie. In this work, the adsorption properties of nanostructured composites prepared by the oxidation of bimetallic Al/Fe nanoparticles with different iron contents were investigated. As a result of oxidation, boehmite AlOOH nanosheets are formed, with the resultant FeAl_2_ nanoparticles being distributed on the surface of boehmite nanosheets. The nanostructured composites prepared from Al/Fe nanoparticles containing 20 wt% Fe have been found to show the highest adsorption capacity towards As(V) specie, being 248 mg/g. The adsorption isotherms are most accurately described by the Freundlich model, with the arsenic adsorption process obeying pseudo second order kinetics. As a result of the study, the optimal ratio of Al and Fe in Al/Fe nanoparticles has been determined to obtain an AlOOH/FeAl_2_ composite adsorbent with a developed and accessible surface and a high sorption capacity towards As(V). This allows us to consider this material as a promising adsorbent for the removal of arsenic compounds from water.

## 1. Introduction

The pollution of groundwater and wastewater with heavy metals is a common environmental threat, as dissolved ions of toxic elements become a risk factor for human health [1,2,3,4]. Arsenic is one of the most abundant contaminants with high toxicity in groundwater worldwide. The presence of arsenic in groundwater requires special attention when assessing the quality of drinking and mineral waters. As groundwater is the main source for drinking water treatment, the removal of arsenic from groundwater is urgently needed.

The most abundant forms, which are commonly found in water, are arsenite (oxidation state +3) and arsenate (oxidation state +5) [4,5]. Removal of As(III) species (arsenite) from contaminated water is a more difficult process than removal of As(V) ones (arsenate). Thus, at pH 6–9, arsenite is mostly in an undissociated form (H_3_AsO_3_), whereas negatively charged anions (H_2_AsO_3_^−^, HAsO_3_^−2^ and AsO_3_^−3^) are only detected at high pH [6,7]. As a result, H_3_AsO_3_ is removed from the water less efficiently, so, commonly, arsenite is oxidized to arsenate beforehand. Due to this, most of the arsenic decontamination technologies are aimed at the efficient removal of arsenate, as it is present at pH values above 2 in the form of H_2_AsO_4_^−^ and HAsO_4_^−2^ anions, which are easy to remove [8,9,10].

Several common methods are used for arsenic removal, which include: ion exchange, coagulation, electrochemical purification, membrane technologies, adsorption, etc. [2,11,12,13,14,15,16,17,18,19]. Adsorption technologies for arsenic wastewater treatment are widely used due to their advantages, such as ease of operation, commercial benefits, etc. Various materials capable of efficient arsenic removal have been described in the literature. Mesoporous aluminum oxide with a specific surface area of 307 m^2^/g and a uniform pore size of 3.5 nm has been shown to uptake 121 mg/g As(V) and 47 mg/g As(III), respectively [20], being stable in the pH range of 3–7. Nanocrystalline TiO_2_ has been found to be also an effective arsenic adsorbent due to its high specific surface area and high affinity for arsenate [21]. Cerium oxide with a specific surface area of 198 m^2^/g exhibited a high affinity for both As(V) and As(III) [22], with the sorption capacities in neutral medium reaching 107 mg/g and 170 mg/g, respectively.

Iron oxides also have a high affinity for arsenic, which makes them the most promising arsenic adsorbents [23,24,25,26]. However, a number of disadvantages, such as low specific surface area or diffusion limitations, contribute to the need for the development of new composite materials for the removal of arsenic from water [27,28,29,30]. Thus, materials based on active carbon/magnetite Fe_3_O_4_ were investigated [27,28,29]. These materials have a specific surface area of up to 1240 m^2^/g, with the maximum adsorption capacity varying from 32.57 mg/g [28] to 455 mg/g [27]. The adsorption characteristics of AlOOH/AlFe composite material towards As(V) were recently investigated [30]. The high specific surface area of 247 m^2^/g and the mesoporous structure of the material are responsible for its adsorption capacity being more than 200 mg/g As(V).

Previously, a material representing bimetallic Al/Fe nanoparticles with 10 wt.% Fe content has been reported, and the influence of the conditions of oxidation with water on the morphology of the resulting nanostructures and their adsorption characteristics with respect to arsenic (V) specie relations was studied [31]. Nanosheet structures obtained by the oxidation of Al/Fe particles with water at 60 °C have been shown to have a higher adsorption capacity than platelet-like and rod-like nanostructures obtained under hydrothermal conditions and in humid air, respectively. The obtained nanosheet structures are porous, with a specific surface area of about 330 m^2^/g and a positive zeta potential, which contributes to the electrostatic attraction of arsenic species in an anionic form. In comparison, aluminum oxide itself, obtained by the oxidation of electro-explosion Al nanoparticles by water, is able to adsorb up to 75 mg/g As(V).

In the present work, the influence of AlFe_2_ content in AlOOH/AlFe_2_ composites on the sorption characteristics towards arsenic was considered for the first time. The optimal Al/Fe ratio in the nanoparticles in order to obtain an AlOOH/AlFe_2_ composite with high sorption capacity towards As(V) has been established.

## 2. Materials and Methods

Bimetallic Al/Fe nanoparticles were obtained by electrical explosion of twisted wires (EETW) of the corresponding metals in argon medium according to the technique reported previously [32]. The EETW method is based on the destruction of a metal wire subjected to a high-current pulse with a current density in the range 10^6^–10^9^ A/cm^2^. The wire heats up and then explodes, giving off metal vapor and liquid metal droplets forming nanoparticles on cooling. The common method for EETW is to use an LC circuit to set the required energy input into the wire.

Table 1 presents the electrical explosion parameters to obtain Al/Fe composites.

Nanostructured adsorbents under study were prepared by oxidation of bimetallic Al/Fe nanoparticles with water at 60 °C for 1 h, according to the technique reported previously [31].

XRD phase identification analyses of nanostructured materials under study were carried out using an XRD 6000 diffractometer (Shimadzu, Kyoto, Japan) in 2θ in the area of 10–90° at 25 °C by using CuKα (λ = 1.54 Å) radiation. XRD qualitative phase analysis was performed with the Powder diffraction file (PDF) database PDF-2 Release 2014.

The crystalline structure, phase composition and morphology of the nanostructured materials were studied by the transmission electron microscopy technique using a JEM-2100 transmission electron microscope integrated with an X-Max energy dispersive spectrometer (Oxford Instruments, Abingdon, UK).

Zeta potential measurements were performed in deionized water at 25 °C and various pH values using a Zetasizer Nano ZSP instrument equipped with an auto-titration unit, MPT-2 (Malvern Instruments Ltd., GB, Malvern, UK), with the use of Zetasizer Software v7.11. For this measurement, a 20 mg powder weight was placed in 10 mL of deionized water and treated in an ultrasonic bath (VU-09-YA-FP-0.3, Ferroplast, Yaroslavl, Russia) for 30 s. Then, 1 mL aliquot was taken and placed in the U-shaped cuvette of the instrument and measured in automatic mode.

Specific surface area and porous structure of the nanostructured materials under study were determined by Brunauer–Emmett–Teller (BET) nitrogen adsorption/desorption method using a Sorbtometer M specific surface analyzer (Katakon, Novosibirsk, Russia).

For investigation of adsorption kinetics, a 50 mg weight of nanostructured composite adsorbent was placed in sodium arsenate solution with a concentration of 400 mg/L and dwelled for different time intervals under continuous stirring. The mixtures were then centrifuged at 3500 rpm for 10 min and the arsenic concentration in the supernatant was measured. The arsenic concentration was determined by the inversion voltammetry method by first reducing As(V) to As(III). Measurement was performed using a voltametric analyzer, TA-Lab (TomAnalit, Tomsk, Russia), with an electrochemical measurement system including gold-carbon working and silver chloride reference electrodes. The background electrolyte was Trilon B solution (0.01 mol/L) with the limit of detection being 0.5 µg/L. The amount of adsorbed arsenic specie for a certain period of time was calculated by Equation (1):

(1) $$q_{t}=\frac{\left(C_{0}-C_{t}\right)*V}{m}$$

where *q_t_* (mg/g) is the amount of As(V) adsorbed per mass of sorbent at any time, *C*_0_ and *C_t_* (mg/L) are the concentrations of As(V) at initial and any time *t*, respectively, *V* is the volume of the solution (L) and *m* is the adsorbent weight (g).

Sodium arsenate stock solution (500 mg/L) has been prepared and the sodium arsenate solutions with the desired concentration were prepared by serial dilution of the stock solution to determine an adsorption isotherm. Then, 50 mg nanostructured composite adsorbent weights were placed in 50 mL sodium arsenate solutions with different concentrations. Adsorption experiment was carried out under continuous stirring for 60 min. Then, the solutions were centrifuged at 3500 rpm for 10 min and measurements were carried out. Adsorption capacity *q_e_* (mg/g) was calculated by Equation (2):

(2) $$q_{e}=\frac{\left(C_{0}-C_{e}\right)*V}{m}$$

where *q_e_* (mg/g) is the amount of As(V) adsorbed per mass of sorbent at equilibrium, *V* (L) is the volume of the solution, *C*_0_ and *C_e_* (mg/L) are initial and equilibrium concentrations of the solute, respectively, and *m* (g) is adsorbent weight.

## 3. Results and Discussion

**Figure 1:**
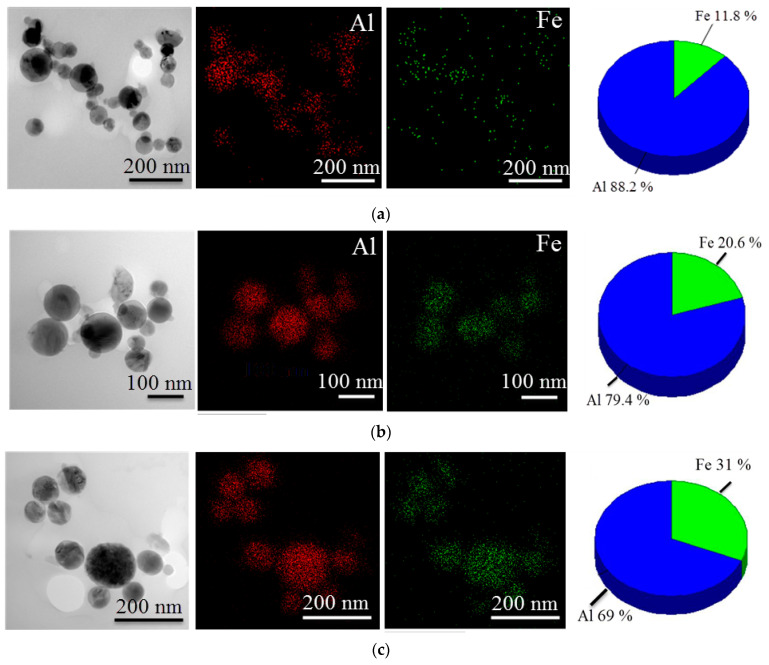
TEM images (left), EDX mapping for elements Al and Fe of initial Al/Fe nanoparticles (center) and elemental percentage (right) for: (**a**)—Al-10Fe; (**b**)—Al-20Fe; (**c**)—Al-30Fe.

According to the TEM/EDX data presented, Al/Fe nanoparticles are of spherical shape and contain both aluminum and iron (Figure 1a–c). The iron/aluminum ratios in Al-10Fe, Al-20Fe and Al-30Fe particles correspond to the theoretical ones and are 11.8 wt.% [31], 20.6 wt.% and 31.0 wt.%, respectively (Figure 1a–c, pie charts). As can be seen from the images, aluminum and iron are distributed throughout the particles. According to the phase diagram of the Al-Fe system [33], Al is well dissolved in α-Fe, forming wide regions of solid solutions with a BCC structure. Due to the fact that nanoparticles in EETW can be formed from both gas and liquid phases [32], the presence of both metals in individual nanoparticles is quite natural.

**Figure 2:**
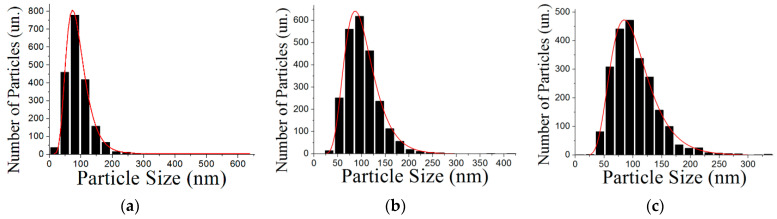
Histograms of the nanoparticle size distribution for: (**a**)—Al-10Fe; (**b**)—Al-20Fe; (**c**)—Al-30Fe.

Al/Fe powders have been found to obey the log-normal particle size distribution (Figure 2). The average sizes of the nanoparticles were determined to be (85 ± 1) nm, (97 ± 1) nm, (97 ± 1) nm for Al-10Fe, Al-20Fe and Al-30Fe, respectively.

**Figure 3:**
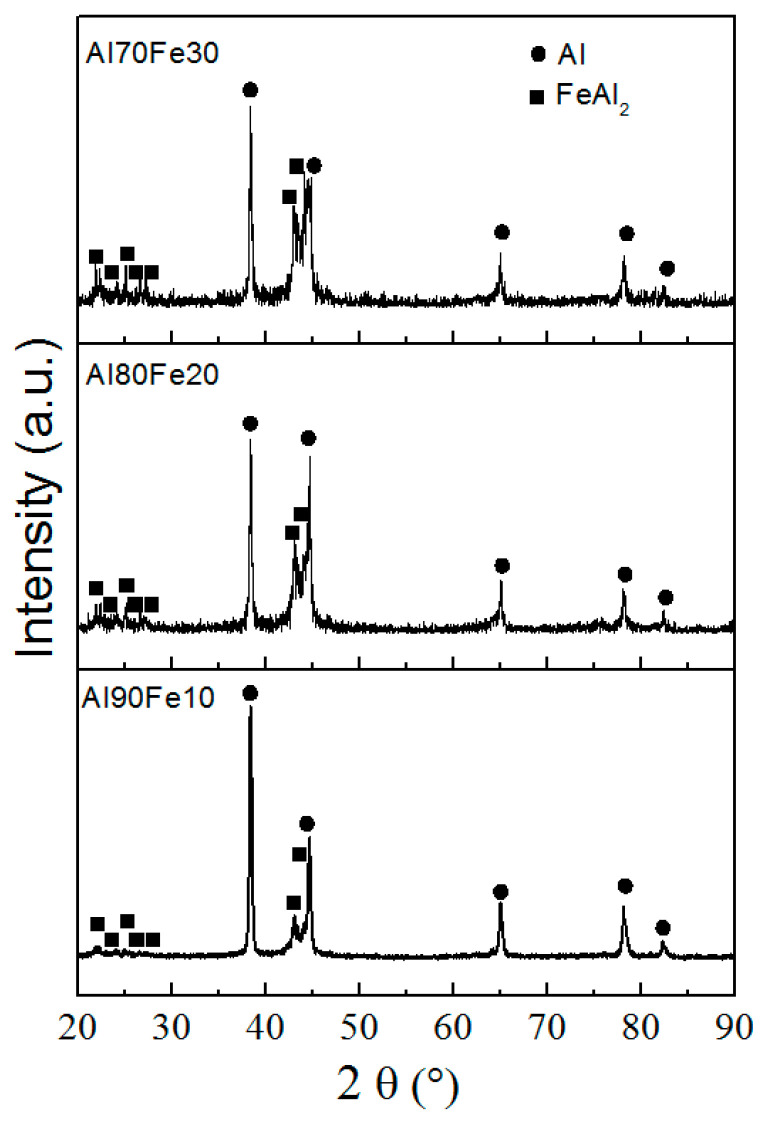
XRD phase analysis patterns of Al/Fe nanopowders.

XRD phase analysis shows aluminum and iron in the Al/Fe nanoparticles presenting in the form of aluminum metal with a standard lattice parameter (4.049 Å) and FeAl_2_ intermetallide, respectively (Figure 3). With increasing iron content in the Al/Fe nanoparticles, the intensity of the FeAl_2_ intermetallide peaks increases.

**Figure 4:**
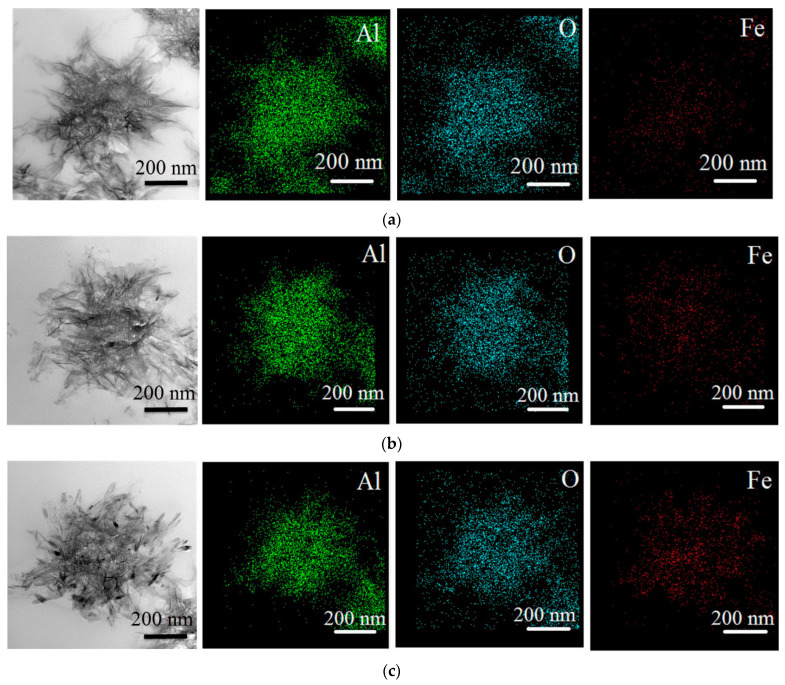
TEM images and EDX mapping for elements Al, O and Fe of the Al/Fe nanoparticle oxidation products: (**a**)—Al-10Fe; (**b**)—Al-20Fe; (**c**)—Al-30Fe.

When oxidized with water, Al/Fe nanopowders form boehmite nanosheets 2–5 nm thick and planar sizes up to 200 nm assembled into agglomerates up to 2 μm in size (Figure 4). The boehmite nanosheet structures are formed also when water oxidizes Al nanoparticles obtained by the electrical explosion of Al wire [34]. In modern concepts, the oxidation of aluminum with water proceeds by an electrochemical mechanism, wherein aluminum acts as an anode, and intercrystalline defects can serve as a cathode. A reduction of water with the formation of hydroxide ions and the release of hydrogen takes place at the cathode. At the anode proceeds the oxidation of aluminum, which begins with the destruction of its crystal lattice and ends with the release of Al^3+^ simple or complex hydrated ions into solution [34]. In weakly alkaline medium are formed monomers [Al(OH)_3_(OH_2_)_3_] giving [A1_4_(OH)_12_(OH_2_)_5_] seeds, which are transformed into distorted tetramers, [Al_4_O(OH)_10_(OH_2_)_5_], yielding in the boehmite nanosheet structures.

According to TEM-EDX data, iron species are uniformly distributed on the nanosheet surface. With increasing iron content, the appearance of opaque inclusions of 10–20 nm in size is observed in the oxidation reaction products (Figure 4c).

**Figure 5:**
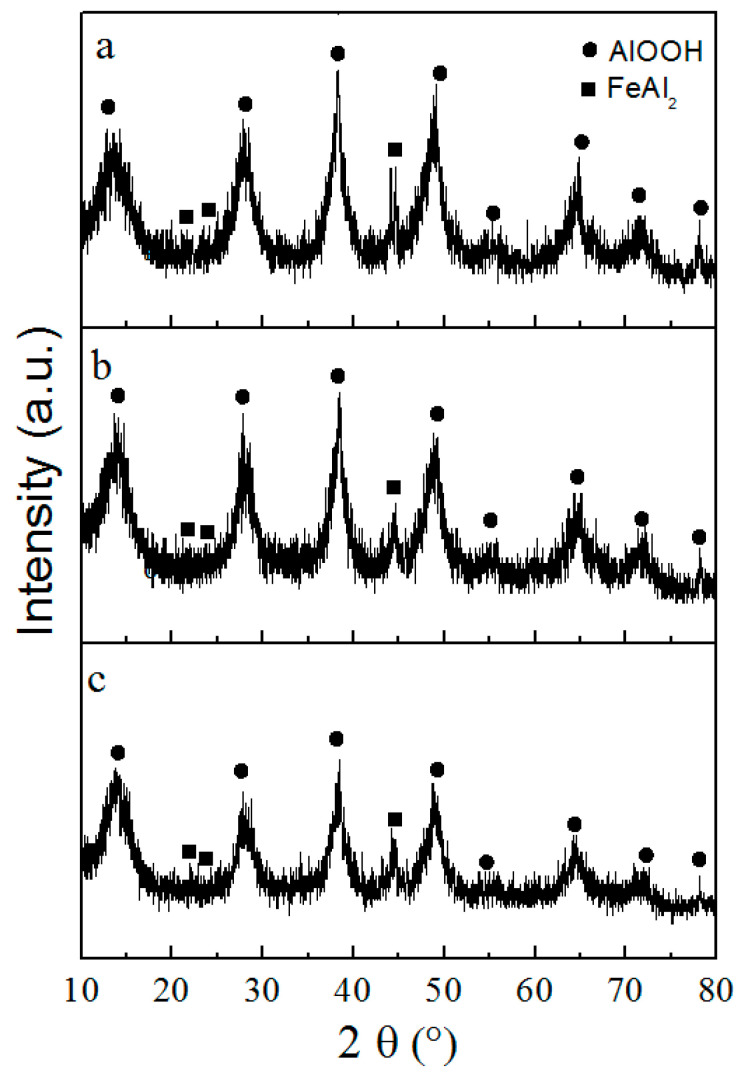
XRD patterns of the Al/Fe nanoparticle oxidation products for: (**a**)—Al-10Fe; (**b**)—Al-20Fe; (**c**)—Al-30Fe.

Broadened peaks characteristic of fine crystalline boehmite are observed in XRD patterns of nanosheet agglomerates [35], and peaks characteristic of FeAl_2_ intermetallide are also identified (Figure 5).

The obtained results indicate that FeAl_2_ intermetallide within nanoparticles does not react with water and is located on the boehmite nanosheet surface. Probably, during the formation of boehmite nanosheets, intermetallide particles released after the dissolution of reacting aluminum become located in the nanosheet agglomerates. Also, with increasing iron content in the Al/Fe nanoparticles, the intermetallide particles located on the boehmite nanosheet surface are enlarged.

**Figure 6:**
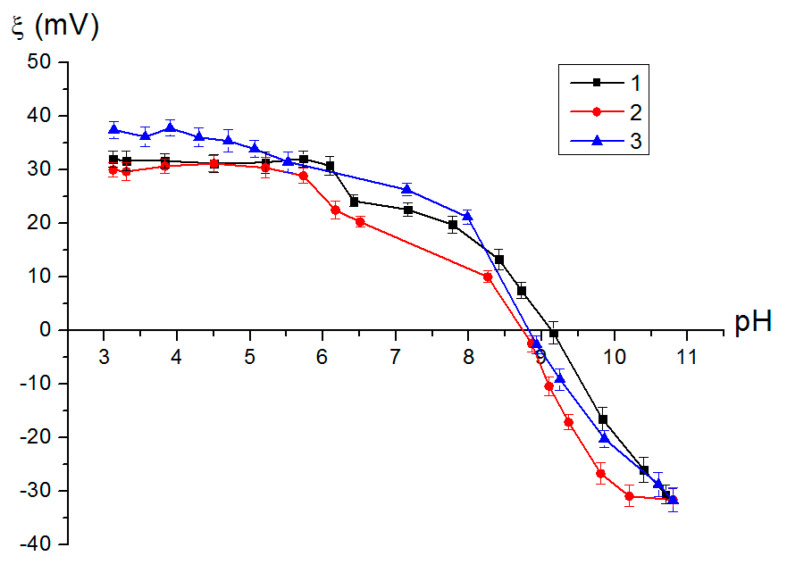
Dependence of the zeta potential on the pH values for the nanostructured composite adsorbents: 1—AlOOH/FeAl_2_ (10%); 2—AlOOH/FeAl_2_ (20%); 3—AlOOH/FeAl_2_ (30%).

One of an adsorbent’s important characteristics is a surface charge, which can be evaluated through the zeta potential of particles. The nanostructured composite adsorbents have been found to show a positive zeta potential in the range of a pH from 3 to 9, which should contribute to the electrostatic interaction of the nanostructured composite adsorbent with arsenic anions. At the same time, with increasing Fe content in the adsorbent samples, no significant changes in the character of the curves or changes in the point of zero charge value were observed (Figure 6). The pH of the point of zero charge for AlOOH/FeAl_2_ (10%) is 9.15, for AlOOH/FeAl_2_ (20%) it is 8.75 and for AlOOH/FeAl_2_ (30%) it is 8.83.

The positive surface charge of the nanostructures in a wide pH range can promote the adsorption of arsenic anionic species due to electrostatic interaction.

**Figure 7:**
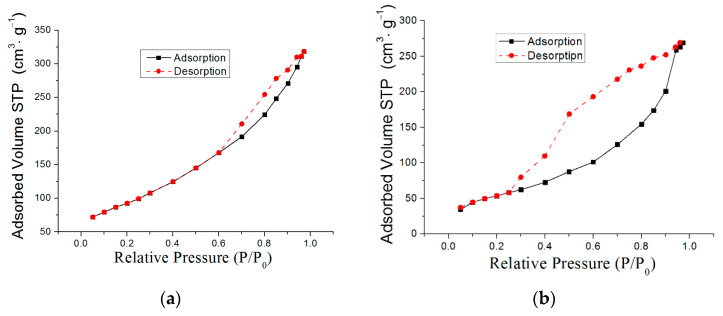

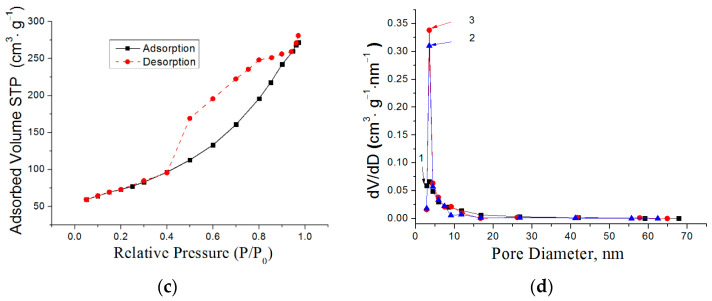
Nitrogen adsorption–desorption isotherms for the nanostructured composite adsorbents AlOOH/FeAl_2_ (10%) (**a**) [31], AlOOH/FeAl_2_ (20%) (**b**), AlOOH/FeAl_2_ (30%) (**c**) and pore size distributions (**d**): 1—AlOOH/FeAl_2_ (10%); 2—AlOOH/FeAl_2_ (20%); 3—AlOOH/FeAl_2_ (30%).

Figure 7a–c show low-temperature nitrogen adsorption–desorption isotherms for the nanostructured composite adsorbents prepared. The isotherms are of type IV, according to the IUPAC classification [36,37], with a pronounced hysteresis loop, which characterizes slit shape pores in the adsorbent samples. Increasing the iron content in composite adsorbent samples leads to a decrease in the specific surface: for AlOOH/FeAl_2_ (10%), the value of the specific surface is 330 m^2^/g, for AlOOH/FeAl_2_ (20%)—282 m^2^/g and for AlOOH/FeAl_2_ (30%)—255 m^2^/g. Pore size distributions for all composite adsorbent samples (Figure 7d) are characterized by pronounced maxima in the region of 4 nm.

**Figure 8:**
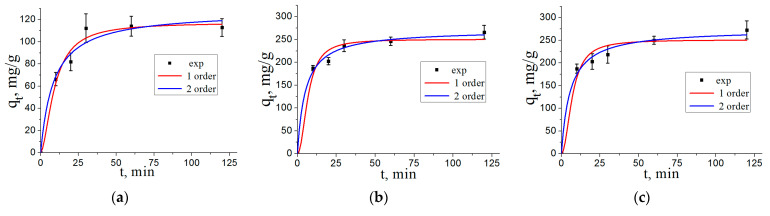
Adsorption kinetics curves of As(V) on the nanostructured composite adsorbents (pH 7.0, initial As(V) concentration 400 mg/L): (**a**)—AlOOH/FeAl_2_ (10%); (**b**)—AlOOH/FeAl_2_ (20%); (**c**)—AlOOH/FeAl_2_ (30%).

The adsorption of arsenate on the nanostructured composite adsorbents as a function of contact time is shown in Figure 8. Adsorption proceeds rather fast and reaches equilibrium in about 60 min for all synthesized adsorbent samples (Figure 8).

As a rule, pseudo first and pseudo second order kinetic models of adsorption kinetics are used to describe adsorption kinetics [38,39]. The pseudo first order model is expressed by Equation (3):

(3) $$\frac{dq}{\;dt}=k\left(q_{e}-q_{t}\right)$$

The integration of Equation (3) takes into account boundary conditions (*q_t_* = 0 for *t* = 0, *q_t_* = *q* for *t* = *t)* and returns Expression (4):

(4) $$q_{t}=q_{e}\left(1-e^{-kt}\right)$$

The pseudo second order model is expressed by Equation (5)

(5) $$\frac{dq}{dt}=k{\left(q_{e}-q_{t}\right)}^{2}$$

The integration of Equation (5) takes into account boundary conditions, and returns Expression (6):

(6) $$q_{t}=\frac{q_{e}^{2}kt}{1+q_{e}kt}$$

The linear forms of the pseudo first and pseudo second order kinetics equations have the form:

*ln*(*q_e_* − *q_t_*) = *lnq_e_* − *k*_1_*t* (7)

and

(8) $$\frac{t}{q_{t}}=\frac{1}{k_{2}q_{e}^{2}}+\frac{1}{q_{e}}t$$

respectively.

**Figure 9:**
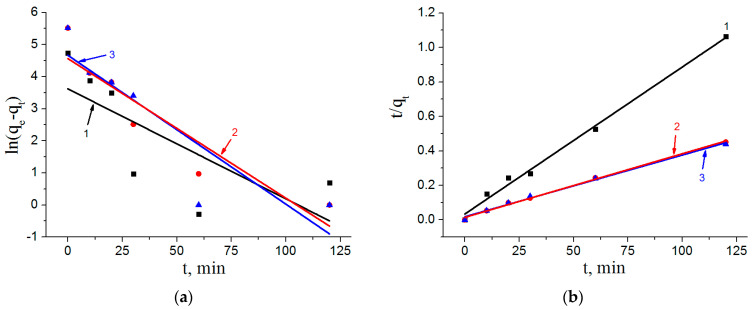
The linear forms of the pseudo first (**a**) and pseudo second order adsorption kinetic models (**b**) kinetic models for the nanostructured composite adsorbents: 1—AlOOH/FeAl_2_ (10%); 2—AlOOH/FeAl_2_ (20%); 3—AlOOH/FeAl_2_ (30%).

The kinetic parameters of arsenate adsorption on the nanostructured composite adsorbents fitted according to the pseudo first order and pseudo second order models are presented in Table 2. Figure 9 shows the plots of the linear forms of pseudo first and pseudo second order kinetic models for the nanostructured composite adsorbents.

According to the values of determination coefficients *R*^2^ for the fitted kinetic parameters, the adsorption of arsenate on the nanostructured composite adsorbents can be assumed to be better described by the pseudo second order model equation. This indicates that the adsorption rate is determined more by the availability of adsorption centers on the adsorbent surface than by the concentration of solute. At the same time, different iron content in the samples does not noticeably affect the adsorption rate.

**Figure 10:**
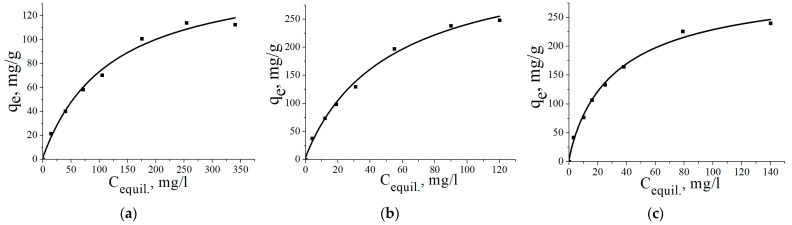
Adsorption isotherms of As(V) specie on the nanostructured composite adsorbents: (**a**)—AlOOH/FeAl_2_ (10%); (**b**)—AlOOH/FeAl_2_ (20%); (**c**)—AlOOH/FeAl_2_ (30%).

The adsorption isotherms of arsenate are shown in Figure 10. As can be seen, the adsorption isotherm curves show similar shapes, which indicates the same adsorption mechanism for all composite adsorbents. An increase in iron content in the nanostructured composite adsorbents from 10 to 20 wt.% leads to an increase in adsorption capacity from 112 mg/g to 248 mg/g, and a further increase in iron content, up to 30 wt.%, practically does not affect adsorption capacity, being 240 mg/g for the AlOOH/FeAl_2_ (30%) adsorbent. This may be due to the formation of larger iron entities during the oxidation of Al-30Fe powder with water.

The adsorption isotherms were analyzed using Langmuir and Freundlich adsorption models [40,41,42,43,44]. The applicability of adsorption models was evaluated by comparing determination coefficients *R*^2^ [44].

The Langmuir model assumes a sorbate monolayer on a homogeneous adsorbent surface at a constant temperature, the distribution of the sorbate between two phases being characterized by the equilibrium constant *K_a_*. The Langmuir adsorption isotherm equation is:

(9) $$q_{e}=q_{max}\;\frac{K_{a}C_{e}}{1+K_{a}C_{e}}$$

where *q_e_* (mg/g) is the amount of As(V) sorption, *C_e_* (mg/L) is the concentration at the stage of equilibrium, *K_a_* is the Langmuir constant (L/mg), *q_max_* is the monolayer Langmuir capacity (mg/g). The linear form for the Langmuir isotherm model is expressed as follows:

(10) $$\frac{1}{q_{e}}=\left(\frac{1}{K_{a\;}q_{max\;}}\right)\frac{1}{C_{e}}+\left(\frac{1}{q_{max}}\right)$$

The Freundlich adsorption model assumes the adsorption process occurs on an energetically inhomogeneous surface. The Freundlich adsorption isotherm equation is:

(11) qe=KfCe1n

where *K_f_* (L/mg) is an indicator of the adsorption capacity and 1/*n* is the adsorption intensity, indicating both the relative distribution of energy and the heterogeneity of the adsorbent sites.

The linear equation for the Freundlich isotherm model is expressed as follows:

(12) $$\log q_{e}=\log K_{f}+\frac{1}{n}logC_{e}$$

The fitted values of the adsorption parameters for the Langmuir and Freundlich adsorption models are summarized in Table 3, and these values were used to plot adsorption isotherms.

**Figure 11:**
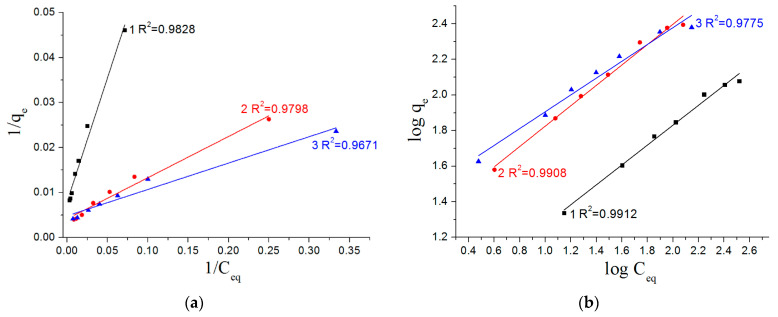
Linear plots of Langmuir (**a**) and Freundlich (**b**) adsorption isotherms for nanostructures: 1—AlOOH/FeAl_2_ (10%); 2—AlOOH/FeAl_2_ (20%); 3—AlOOH/FeAl_2_ (30%).

Figure 11 presents the adsorption isotherms in linear form. Based on the values of determination coefficient *R*^2^, the sorption of As(V) for all types of the nanostructured composite adsorbents is most adequately described by the Freundlich equation. In addition, Freundlich isotherm constant 1/*n* can be used to evaluate the favorability adsorption level and the degree of heterogeneity of the adsorbent surface. The sorption process is irreversible when 1/*n* = 0, favorable adsorption is in the case of 0.1 < 1/*n* < 1, and at 1/*n* > 1—unfavorable condition [45]. As summarized in Table 3, in all cases, the Freundlich isotherm constant values are in the range of 0.1 to 1.

Thus, the increase in the Fe content in the initial Al/Fe nanoparticles, of up to 20 wt.%, allowed for more than a 2-fold increase in the sorption capacity of AlOOH/FeAl_2_ nanostructured composite adsorbents with respect to As(V) specie. A further increase in Fe content, up to 30 wt.%, did not lead to an increase in adsorption capacity, which may be due to the enlargement of iron-containing nanoparticles in the composite adsorbent and the corresponding reduction of the surface’s contribution to the sorption process.

The obtained results on the adsorption of arsenate on the surface of the prepared nanostructures indicate that their adsorption efficiency is mostly determined not by the amount of intermetallide, but by its dispersivity. The increase in iron content, to up to 30 wt.%, leads to the fact that the surface of intermetallide particles in the AlOOH/FeAl_2_ nanostructures does not increase, and consequently, there is no increase in the number of sites responsible for the adsorption of As(V).

One should also note the high adsorption capacity of AlOOH/FeAl_2_ (20%) nanostructures achieved with respect to arsenate, which exceeds many reported arsenic adsorbents [20,21,22,28], which makes the above nanostructures very promising in this direction of use. Boehmite in the AlOOH/FeAl_2_ nanosheet structures promoting arsenic adsorption [31] is a good adsorbent for anionic dyes, negatively charged nanoparticles, and bacteria and viruses [46]. Its efficient adsorption of As(V) on the surface of nanostructured AlOOH, as well as other negatively charged species, is due to its highly developed surface and positive surface charges, which present over a wide pH range (3–9). The positive surface charge of AlOOH favors the electrostatic attraction of negatively charged entities. Clearly, a treatment of composites after the adsorption of As(V) in weakly alkaline solutions will result in their regeneration due to the change of the adsorbent surface charge. This makes the AlOOH/FeAl_2_ nanostructures a universal adsorbent for the purification of contaminated water. Zeta potential measurements suggest that arsenic desorption at a high pH is possible, and the consideration of the regeneration of adsorbents is planned. Also, in the future, we plan to focus on studying the adsorption mechanisms of As compounds and other pollutants by AlOOH/FeAl_2_ (20%) composite.

## 4. Conclusions

The effect of the FeAl_2_ content in AlOOH/FeAl_2_ composites on their adsorption characteristics towards As(V) was studied for the first time.

The oxidation of bimetallic Al/Fe nanoparticles with iron contents of 10–30% by water at 60 °C has been found to yield nanosheet composite nanostructures of fine crystalline boehmite, on the surface of which FeAl_2_ intermetallide particles are distributed. The nanosheet structures so prepared have a positive zeta potential in the acidic and neutral pH ranges and a high specific surface area of 255–330 m^2^/g due to the mesoporous structure of the composites.

When studying the adsorption characteristics of the prepared composite nanostructures towards As(V), the adsorption process has been found to be best described by the Freundlich model, indicating the inhomogeneity of the surface of the nanostructures as well as possible multilayer adsorption. The arsenic adsorption process obeys pseudo second order kinetics.

The adsorption capacity of nanostructures prepared from Al/Fe nanoparticles containing 10 wt.% Fe was 112 mg/g, containing 20 wt.% Fe—248 mg/g, containing 30 wt.% Fe—240 mg/g. The lack of increase in the sorption capacity with respect to As(V) specie for nanostructures prepared from Al/Fe nanoparticles containing 30 wt.% Fe may be due to the enlargement of FeAl_2_ intermetallide particles.

As a result, the optimal ratio of Al and Fe in the Al/Fe nanoparticle composition has been determined to obtain an AlOOH/FeAl_2_ composite with a high adsorption capacity towards As(V). The obtained results will be useful for solving environmental problems related to arsenic removal from water.

## Figures and Tables

**Table 1:** The electrical explosion parameters to obtain Al/Fe composites.

Sample	Wire Diameter, mm		Wire Length, mm	C, μF	U, kV	P, MPa
	Fe	Al				
Al90Fe10	0.1	0.45	90	3.2	31	0.3
Al80Fe20	0.1	0.35	75	2.0	27	0.3
Al70Fe30	0.1	0.25	60	1.2	25	0.3

**Table 2:** Kinetic parameters of arsenic adsorption on the nanostructured composite adsorbents.

Kinetic Parameters	Reaction Order	
	I	II
	AlOOH/FeAl_2_ (10%)	
q_e_, mg/g	114.81	127.57
k	0.0792	0.0009
R^2^	0.5516	0.9935
	AlOOH/FeAl_2_ (20%)	
q_e_, mg/g	248.79	271.11
k	0.1178	0.0007
R^2^	0.8657	0.9969
	AlOOH/FeAl_2_ (30%)	
q_e_, mg/g	248.97	274.35
k	0.1124	0.0006
R^2^	0.8050	0.9948

**Table 3:** Adsorption model parameters.

Samples	BET Surface Areas (m^2^/g)	Langmuir Model			Freundlich Model		
		*q_max_* (mg/g)	*K_a_*	*R^2^*	*K_f_*	1/*n*	*R* ^2^
AlOOH/FeAl_2_ (10%)	330	144.2	0.0107	0.9828	5.51	0.54	0.9912
AlOOH/FeAl_2_ (20%)	282	313.6	0.0275	0.9798	17.99	0.57	0.9908
AlOOH/FeAl_2_ (30%)	255	258.9	0,0494	0.9671	27.21	0.46	0.9775

## Data Availability

The data presented in this study are available on request from the corresponding author.
